# Clinical characteristics, risk factors and prognosis of *Klebsiella pneumoniae* infection in patients with different states of immune function: a retrospective study

**DOI:** 10.3389/fcimb.2025.1539554

**Published:** 2025-05-30

**Authors:** Xin Wang, Chonghe Xu, Chao Qin, Juan Liu, Xiaoli Kong, Zhijun Zhu, Chenchen Zhang, Wei Xu, Mei Zhu

**Affiliations:** ^1^ Department of Clinical Laboratory, The Affiliated Chaohu Hospital of Anhui Medical University, Chaohu, Anhui, China; ^2^ School of Basic Medical Sciences, Capital Medical University, Beijing, China; ^3^ Department of Blood Transfusion, The First Affiliated Hospital of Anhui Medical University, Hefei, Anhui, China

**Keywords:** *Klebsiella pneumoniae*, infection, colonization, clinical characteristics, risk factors

## Abstract

**Background:**

The aim of our study was to investigate the epidemiology and risk factors of *Klebsiella pneumoniae* (KP) infection in immunocompromised patients and to compare the differences in prognosis among patients with different immune states in the extended-spectrum beta-lactamase KP (ESBL-KP), the carbapenem-resistant KP (CRKP), and the non-multidrug-resistant KP (non-MDR-KP) groups.

**Methods:**

We conducted a retrospective study in immunocompromised and immunocompetent patients with KP infections who were admitted to Chaohu Hospital of Anhui Medical University from January 2023 to December 2023. We compared demographics, clinical characteristics, treatments, and outcomes across these groups and examined the impact of ESBL-KP, CRKP and non-MDR-KP on cumulative survival rates in populations with different immune states by plotting Kaplan-Meier curves.

**Results:**

Our study included 228 immunocompromised patients and 200 immunocompetent patients. Compared to the immunocompetent group, immunocompromised patients were more likely to have a history of surgery and to use hormone frequently. They tended to rely more on medical devices, including urinary catheters, nasogastric catheters, arterial catheters, venous catheters, mechanical ventilation and endoscopy. Immunocompromised patients had a poorer recovery and were rehospitalized more often than those in immunocompetent patients. In the multivariable analysis, age-adjusted Charlson comorbidity index (aCCI) (OR: 1.292, 95%CI: 1.086-1.537, *P* < 0.004) and microbiological clearance failure (OR: 4.175, 95%CI: 1.966-8.866, *P* < 0.001) were the most important risk factors for mortality in immunocompromised patients infected with KP. It was further observed that immunocompromised patients in the ESBL-KP group had the lowest cumulative survival. In contrast, among both all participants and immunocompetent patients, the CRKP group had the lowest cumulative survival, followed by the ESBL-KP group.

**Conclusion:**

The clinical characteristics, treatment process and prognosis in immunocompromised patients with KP infections are significantly different from those in immunocompetent patients. In clinical settings, the standardization of invasive procedures and the rational use of antibiotics represent the most effective strategies for preventing and treating KP infection.

## 1 Introduction

*Klebsiella pnenmoniae* (KP) is a prevalent Gram-negative pathogen in hospital environment, which causes a wide range of infections, such as pneumonia, urinary tract infections (UTIs), septicemia, and meningitis (Kain et al., 2024; Wang et al., 2020; Dan et al., 2023). According to the data from CHINET, in 2024, KP constituted 13.9% of the clinical isolates, only second to *Escherichia coli*, exerting tremendous pressure on the medical system in China. The extended-spectrum beta-lactamase KP (ESBL-KP) and the carbapenem-resistant KP (CRKP) have also gradually emerged and spread widely (Mohd Asri et al., 2021). Most studies reported mortality rates reach 33-50% in patients infected with ESBL-KP or CRKP (Çölkesen et al., 2023; Li et al., 2023). Therefore, prevention of KP infection, especially the multidrug-resistant KP (MDR-KP) infection, is important and extremely urgent.

Due to the nature of the disease itself and the use of immunosuppressive treatments, there is an increase in the number of immunocompromised patients. However, existing guidelines on infectious diseases are usually based on immunocompetent populations. The research on risk factors for KP infections, as well as their morbidity and mortality in patients with different immune states, is still limited. Di Pasquale et al. suggested that the causative pathogens, clinical characteristics, treatment, and prognosis of infections in immunocompromised patients with KP in the community differed significantly from those in immunocompetent populations (Di Pasquale et al., 2019). Mohd Asri et al. demonstrated that immunocompromised patients had undergone more invasive procedures during hospitalization than immunocompetent patients (Mohd Asri et al., 2021). Liu et al. on this basis suggested that the neutrophil count and the gamma glutamyl transpeptidase (gGT) level were higher in immunocompromised patients than those in immunocompetent patients. And the levels of creatinine, alanine aminotransferase, aspartate aminotransferase, and gGT changed significantly in both two groups during treatment, suggesting the importance of regularly monitoring the liver and renal functions of patients (Liu et al., 2023).

To treat infections with MDR-KP is a difficult problem. ESBL-KP have the ability to inactivate both penicillins and first-, second- and third-generation cephalosporins (in addition to cephamycins), as well as monobactams (Mączyńska et al., 2023). CRKP are resistant to carbapenemases which are generally considered antibiotics of last resort for KP infections, posing great challenge in therapy. The situation is even worse when the infection targets the elderly, immunocompromised patients or infants with immature immune systems (Farhadi et al., 2021; Tofarides et al., 2023; Sawatwong et al., 2019). Premachandra et al. found that the presence of immunosuppressive diseases or drugs was a risk factor for infection with MDR-KP (Timsit et al., 2019; Premachandra and Moine, 2024). However, to our knowledge, few papers have grouped populations with different immune function states based on the resistance characteristics of the isolates. Therefore, our study aims to examine the clinical characteristics and prognosis of patients with ESBL-KP, patients with CRKP, and patients with the non-multidrug-resistant KP (non-MDR-KP) within populations of different immune states.

The primary objectives of our research were to compare the differences between immunocompromised and immunocompetent individuals after KP infections and to investigate the independent risk factors influencing mortality in the immunocompromised patients. The secondary objective was to investigate differences in clinical characteristics and cumulative survival probabilities among ESBL-KP, CRKP, and non-MDR-KP groups within populations exhibiting different immune function states.

## 2 Methods

### 2.1 Study design

This retrospective study included patients with positive KP cultures in all sample types (sputum, blood, urine, wounds, etc.) who were admitted to Chaohu Hospital of Anhui Medical University from January 2023 to December 2023. Patients with incomplete medical records or those who had not been discharged from the hospital at the time of data collection were excluded. For patients with multiple hospital admissions during the study period, only data from the first hospital admission were included. The study was approved by the Ethical Review Committee of Chaohu Hospital of Anhui Medical University (no: KYXM-202312-053).

### 2.2 Data collection

Through the hospital information system (LIS), we collected the following information about patients: gender, age, department, activities of daily living (ADL) score, age-adjusted Charlson comorbidity index (aCCI), quick sepsis-related organ failure assessment (qSOFA), sample type, presence of bacterial co-infections, alcohol and tobacco consumptions, medical history (surgical operation, hospitalization, infection), comorbidities (nervous system diseases, respiratory diseases, cardiovascular diseases, digestive diseases, history of solid organ tumor, chronic kidney diseases and chronic hepatic diseases), invasive procedures (urinary catheter, nasogastric catheter, T-tube catheter, arterial catheter and venous catheter, mechanical ventilation, tracheal cannula, tracheostomy, endoscopy, bronchoscope, hemodialysis, peritoneal dialysis, etc.), length of stay(LOS), length of stay (LOS) after infection, length of stay in the intensive care unit (ICU), and routine laboratory tests within 24 hours of admission. Key indicators involved in the tests included white blood cell (WBC), neutrophil count (NEUT), monocyte count (MONO), hemoglobin (HGB), platelet count (PLT), lymphocyte count (YLC), neutrophil-to-lymphocyte ratio (NLR), platelet-to-lymphocyte ratio (PLR), alanine aminotransferase (ALT), aspartate aminotransferase (AST) and creatinine (CR). We also recorded the clinical regression, use of hormonal agents, and antimicrobial treatment strategies of patients, and followed up all the patients included in the study.

### 2.3 Microbiological identification methods

The culture of the strains was performed according to the National Clinical Laboratory Procedures of China (4th Edition). Clinical specimens were inoculated into Columbia blood plates, Chocolate plates or Haemophilus chocolate plates, and MacConkey plates, all of which were 9 cm. The samples were incubated in an incubator at 35°C with 5% CO_2_ for 24–36 h. Colony identification was performed by Bruker MALDI-TOF-MS. For drug sensitivity testing, AST-N13 or AST-N335 cards were used with the VITEK-2 automatic bacterial identification and drug sensitivity analyzer (BioMérieux, France). The results were determined according to 2023 Clinical Laboratory Standardization Institute Pharmacovigilance Specification CLSI M100. The quality control strains used were *Escherichia coli* ATCC25922 and *Pseudomonas aeruginosa* ATCC27853.

### 2.4 Definitions

Participants were classified into immunocompetent and immunocompromised groups. Patients were considered immunocompromised if they met at least one of the following criteria: (1) asplenia; (2) active malignancy, or history of cancer chemotherapy or radiotherapy during the last 3 months; (3) HIV infection with a CD4+ lymphocyte count < 200 cells/mL or a percentage < 14%; (4) history of solid organ transplantation or hematopoietic stem cell transplantation; (5) history of corticosteroid therapy with a daily prednisone dose of at least 20 mg or an equivalent for at least 14 days, or a cumulative prednisone dose exceeding 700 mg; (6) receiving biologic modulators; (7) receiving disease-modifying antirheumatic drugs or other immunosuppressive drugs; (8) liver cirrhosis; (9) severe burns; (10) primary immune deficiency diseases or acquired immune deficiency disorders; (11) hematological diseases, including aplastic anemia, lymphoma, multiple myeloma, acute or chronic leukemia; and (12) neutropenia, defined as having a neutrophil count of less than 1.5 × 10^9^/liter (Solomou et al., 2021; Liu et al., 2023).

A neoplastic disease was defined as active if it required medical or surgical intervention within the last year or if non-treatable metastases were present at the time of study enrollment. In an appropriate antimicrobial strategy, empirical therapy was defined as the antibiotics administered before a susceptibility report was available, and combination therapy was defined as the administration of more than one antibiotic (Xu et al., 2023). The diagnostic criteria for KP infection include: (1) A KP-positive result from a clinically relevant sterile site; (2) respiratory tract infections, defined according to the “*Diagnostic Criteria for Hospital-Acquired Pneumonia and Ventilator-Associated Pneumonia in Chinese Adults*” (2018 Edition) and the “*Guidelines for the Diagnosis and Treatment of Adult Community-Acquired Pneumonia*” (2018 Practical Edition). (3) urinary tract infections, based on the “*Chinese expert consensus on the diagnosis and treatment of urinary tract infections*” (2015 Edition) (Guo et al., 2024; Zhang et al., 2025). Often, patients with KP identified from clinical cultures but who do not meet the above infection criteria, they will be considered for colonization (Howard-Anderson et al., 2022). A hospital-acquired infection (HAI) was characterized by the first positive culture obtained 48 h or more after hospital admission and no evidence of infection at admission. If the infection was directly related to urinary catheters, arterial catheters, and venous catheters, blood transfusion, or surgical operation, it was defined as a healthcare-associated infection (HCAI), regardless of whether the identification time was 48 h after admission. A community-acquired infection (CAI) was characterized by the first positive culture obtained less than 48 h after hospital admission (Haque et al., 2020; Li et al., 2023). A co-infection was characterized by KP, and other microbial species detected in the same specimen (Sophonsri et al., 2023). KP is classified as CRKP if its MIC for imipenem or meropenem is 4 mg/L or higher. It is designated as ESBL-KP if the MIC for ceftazidime, ceftriaxone, cefotaxime, or aztreonam is 2 mg/L or higher (Wang et al., 2023). MDR-KP is characterized by resistance to more than three classes of antibiotics. Microbiological clearance was defined as eradication of the original causative organism from subsequent cultures with 14 days after initiation of treatment (Hu et al., 2024). Clinical outcomes were assessed through patient discharge records, and the 30-day mortality was defined as deaths within 30 days after the onset of KP infection.

### 2.5 Statistical analysis

Categorical variables are described as frequencies (percentages), while continuous variables are presented as mean and standard error of the mean (SEM) values for normally distributed data or as median and interquartile range (IQR) values for data not normally distributed (Kolmogorov-Smirnov test). Categorical variables were analyzed using the chi-square test or the Fisher’s exact test, as appropriate. Continuous variables were analyzed using the T test for normally distributed data, or the non-parametric Mann-Whitney U test after verifying a nonnormal distribution.

We compared the clinical characteristics of immunocompetent and immunocompromised patients and identified independent risk factors affecting mortality in immunocompromised patients through univariate and multivariate logistic regression analyses. Variables with *P* < 0.05 in the univariate analysis were included in the subsequent multivariate logistic regression models. Results were summarized as odds ratios (ORs) with 95% confidence intervals (CIs). All statistical tests were performed using a two-tailed test, with *P* < 0.05 indicating statistical significance. Based on the resistance characteristics of KP, we divided the total, immunocompromised, and immunocompetent populations included in this study into three groups, respectively. If a significant difference was detected among the three groups, we employed Bonferroni’s correction to identify which pairs of groups exhibited statistically significant differences. In such analyses, *P* < 0.017 was deemed statistically significant. In addition, Kaplan-Meier curves were applied to compare the cumulative survival rates of the three groups. The analysis was completed using the SPSS version 25.0.0.2 statistical package (IBM Corporation, Armonk, NY, USA). The results were visualized using OriginPro version 2021 (OriginLab Corporation, Northampton, MA, USA).

## 3 Results

### 3.1 Baseline characteristics

**Figure 1:**
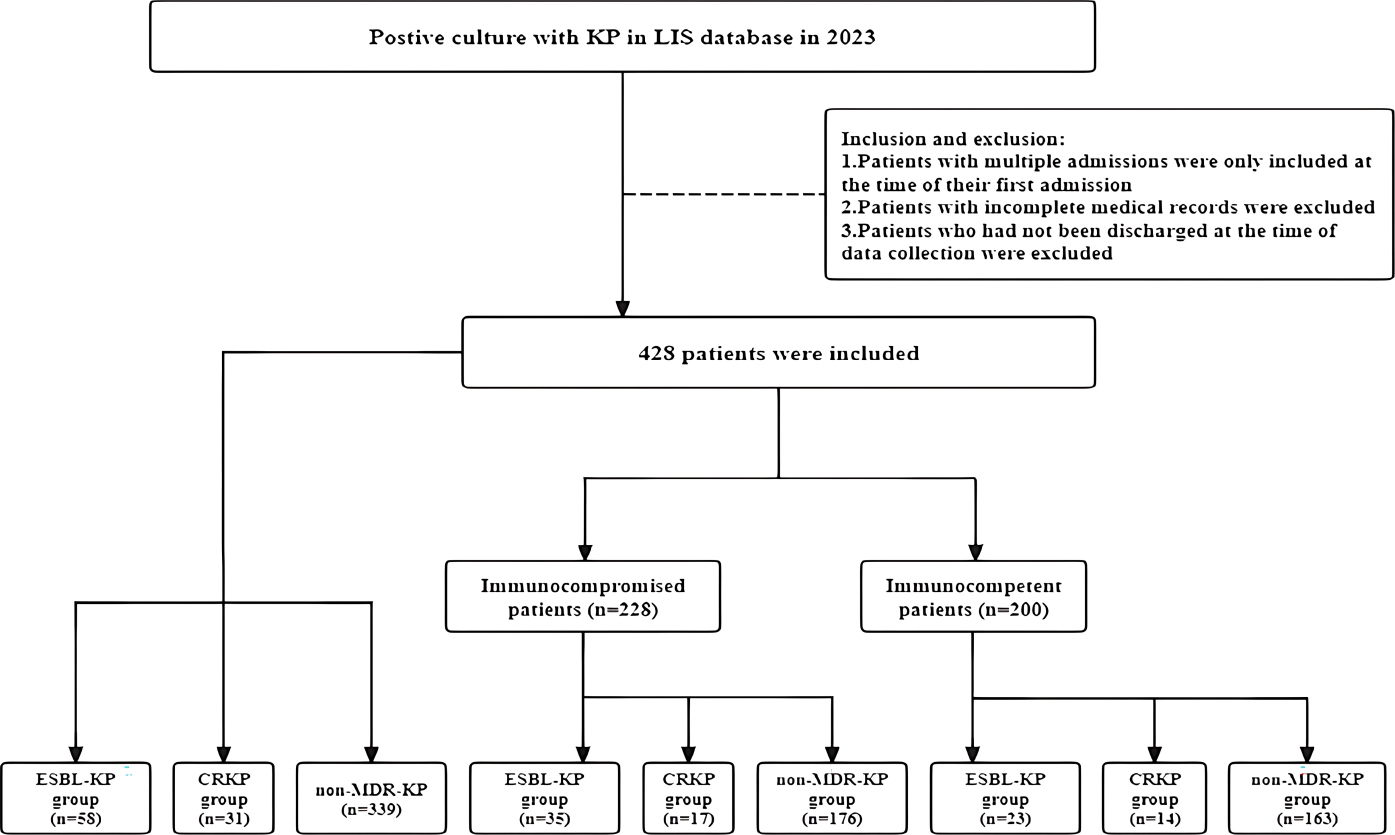
Flowchart of the patients included in the study.

**Figure 2:**
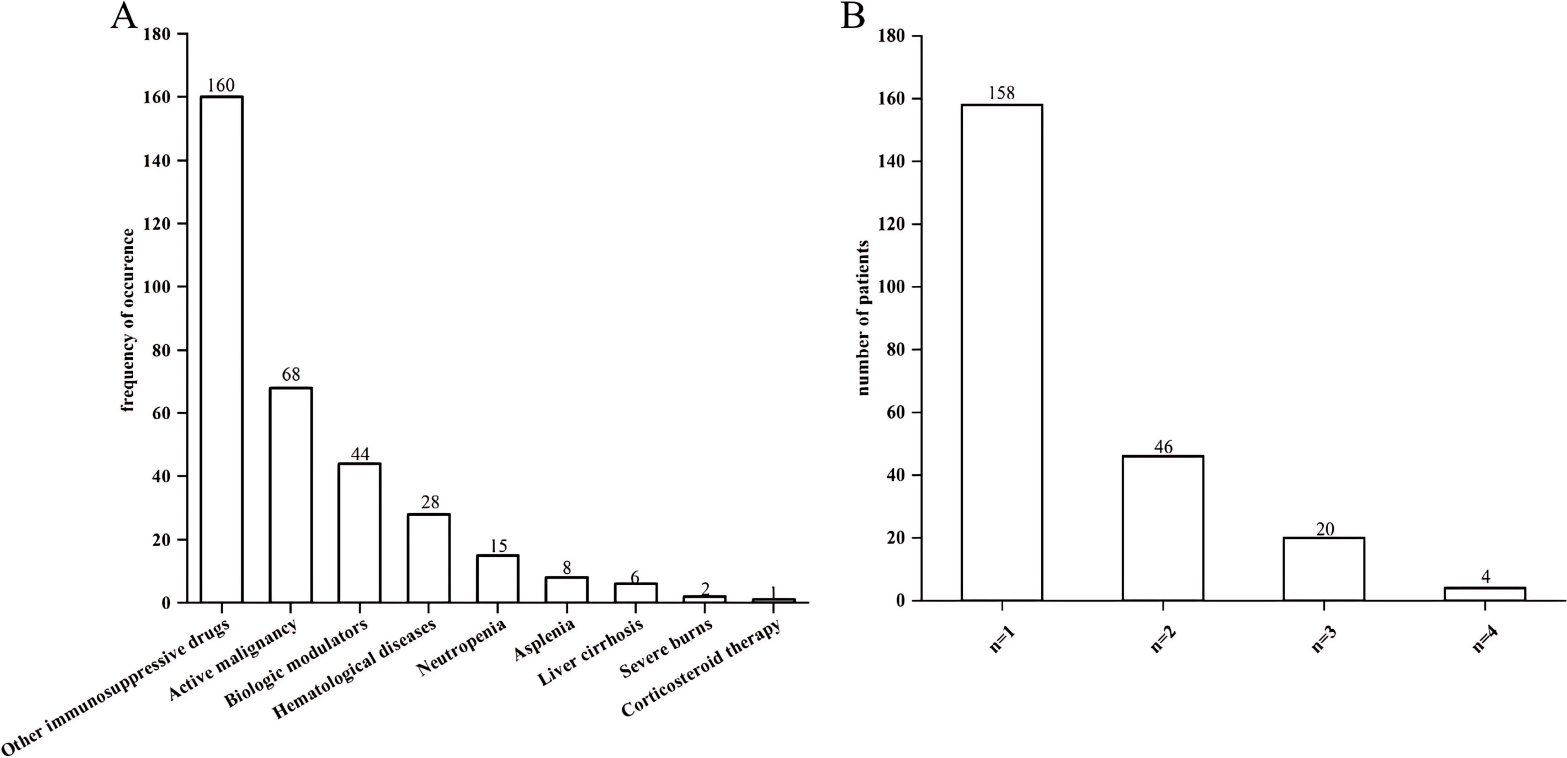
Bar charts of underlying conditions in immunocompromised patients. **(A)** Prevalence of each single underlying condition for immunocompromise of KP infection. **(B)** Prevalence of the number of underlying condition present simultaneously in a single patient.

**Figure 3:**
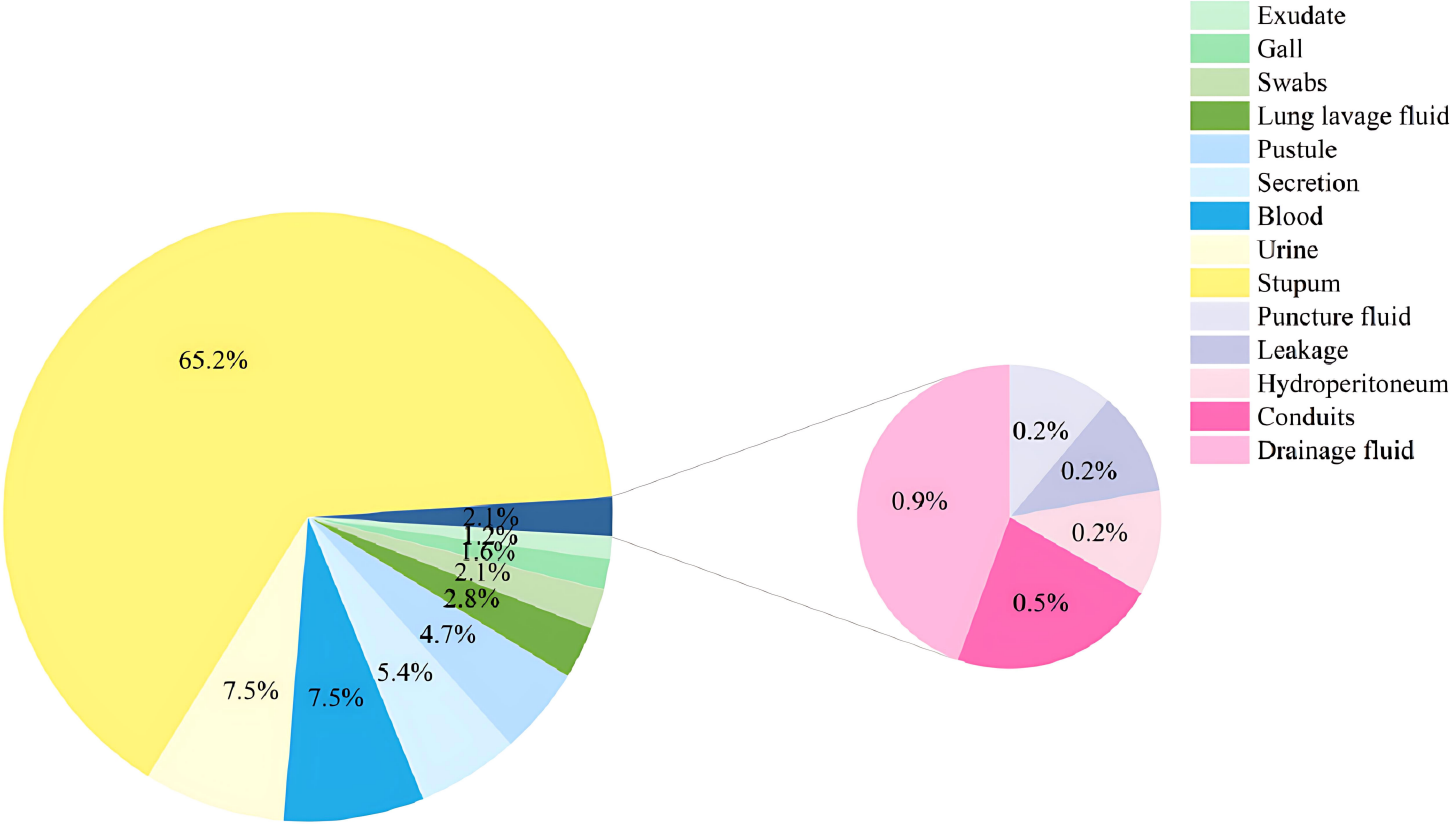
Distribution of KP sample types in the hospital.

Of all the 428 patients involved with KP infections, 228 (53.27%) were immunocompromised, and 200 (46.73%) were immunocompetent (**Figure 1**). The prevalence of each underlying condition for being immunocompromised is depicted in **Figure 2A**, with the use of immunosuppressive drug (70.18%) and active malignancy (29.82%) being the most frequent underlying conditions. A total of 70 patients had more than one underlying condition for being immunocompromised (**Figure 2B**). And when it comes to the foci of infection, the main source of infection in all patients was the respiratory tract (68.0%), with KP found in sputum and lung lavage fluid samples, followed by the urinary tract (7.5%) and bloodstream (7.5%) (**Figure 3**). The rate of respiratory infections in immunocompromised patients exceeded that of immunocompetent patients (73.25% vs. 62%, *P* = 0.013). Within the 428 isolates, the drug resistance rate of KP to ampicillin/sulbactam was highest (26.87%). And our study found that the resistance rates of all drugs were higher in immunocompetent patients than those in immunocompromised patients (**Table 1**).

**Table 1:** The drug resistance rate of KP in all patients.

Variables	Total (n=428)	Immunocompromised patients (n=228)	Immunocompetent patients (n=200)	*P*-value*
AMK	31 (7.24%)	18 (7.89%)	13 (6.50%)	0.579
ATM	85 (19.86%)	50 (21.93%)	35 (17.50%)	0.252
CAZ	74 (17.29%)	42 (18.42%)	32 (16.00%)	0.509
CIP	98 (22.90%)	56 (24.56%)	42 (26.00%)	0.382
CRO	93 (21.73%)	54 (23.68%)	39 (19.50%)	0.295
CTT	32 (7.48%)	17 (7.46%)	15 (7.50%)	0.986
CZO	106 (24.77%)	63 (27.63%)	43 (21.50%)	0.143
FEP	51 (11.92%)	29 (12.72%)	22 (11.00%)	0.584
GEN	64 (14.95%)	37 (16.23%)	27 (13.50%)	0.430
IPM	29 (6.78%)	16 (7.02%)	13 (6.50%)	0.832
LVX	78 (18.22%)	46 (20.18%)	32 (16.00%)	0.264
SAM	115 (26.87%)	65 (28.51%)	50 (25.00%)	0.414
TOB	70 (16.36%)	42 (18.42%)	28 (14.00%)	0.217
TZP	44 (10.28%)	26 (11.40%)	18 (9.00%)	0.414

*:*P* < 0.05. AMK, amikacin; ATM, amitranam; CAZ, ceftazidime; CIP, ciprofloxacin; CRO, ceftriaxone; CTT, cefotetan; CZO, cefazolin; FEP, cefepime; GEN, gentamicin mikacin; IPM, imipenem; LVX, levofloxacin; SAM, ampicillin/sulbactam; SXT, Trimethoprim/Sulfamethoxazole; TOB, tobramycin; TZP, piperacillin/tazobactam.

Baseline characteristics of immunocompetent and immunocompromised patients are shown in **Table 2**. There were no differences in gender, age, medical history (alcohol and tobacco consumption, hospitalization and infection), ADL, qSOFA, bacteremia, or shock between the groups. More immunocompromised patients had fever at discharge (*P* = 0.014) and surgery within 3 months prior to admission (*P* = 0.002) compared to immunocompetent patients. Additionally, more patients in the immunocompromised group had comorbidities, such as history of solid organ tumors (*P* = 0.007). Therefore, their aCCI scores were higher (*P <* 0.001). Healthcare-associated infections were more common in immunocompromised patients (*P* < 0.001), while community-acquired infections were observed more in immunocompetent patients (*P* < 0.001).

**Table 2:** Baseline. epidemiological characteristics, microbiological characteristics, invasive manoeuvres, antimicrobial strategies and clinical outcomes (immunocompromised vs. immunocompetent).

Variables	Total (n=428)	Immunocompromisedpatients (n=228)	Immunocompetentpatients (n=200)	*P*-value*
**Sex, M**	302 (70.56%)	161 (70.61%)	141 (70.50%)	0.979
Age group, y				
0-18	6 (1.40%)	5 (2.19%)	1 (0.50%)	0.137
19-45	32 (7.48%)	13 (5.70%)	19 (9.50%)	0.136
46-65	125 (29.21%)	60 (26.32%)	65 (32.50%)	0.160
>65	265 (61.92%)	150 (65.79%)	115 (57.50%)	0.078
Fever ≥ 72H				
Time at admission	72 (16.82%)	45 (19.74%)	27 (13.50%)	0.085
Time at discharge	23 (5.37%)	18 (7.89%)	5 (2.50%)	**0.014**
No more fever after treatment	56 (13.08%)	32 (14.04%)	24 (12.00%)	0.533
History				
Drinking	43 (10.05%)	19 (8.33%)	24 (12.00%)	0.457
Smoking	46 (10.75%)	29 (12.72%)	17 (8.50%)	0.160
Surgical operation	188 (43.93%)	116 (50.88%)	72 (36.00%)	**0.002**
Hospitalization	250 (58.41%)	140 (61.40%)	110 (55.00%)	0.180
Infection	286 (66.82%)	158 (69.30%)	128(64.00%)	0.245
Scores				
ADL	60 (20, 90)	60 (20, 90)	60 (20, 90)	0.819
aCCI	2 (4, 5)	4 (2.25, 6)	3 (2, 4)	**<0.001**
qSOFA				
0	293 (68.46%)	163 (71.49%)	130 (65.00%)	0.149
1	119 (27.80%)	58 (25.44%)	61 (30.50%)	0.244
2	14 (3.27%)	5 (2.19%)	9 (4.50%)	0.181
3	2 (0.47%)	2 (0.88%)	0 (0.00%)	0.184
Comorbid conditions				
Nervous system diseases	146 (34.11%)	71 (31.14%)	75 (37.50%)	0.166
Respiratory diseases	199 (46.50%)	116 (50.88%)	83 (41.50%)	0.052
Cardiovascular diseases	188 (43.93%)	92 (40.35%)	96 (48.00%)	0.112
Digestive diseases	73 (17.06%)	44 (19.30%)	29 (14.50%)	0.188
History of solid organ tumor	12 (2.80%)	11 (4.82%)	1 (0.50%)	**0.007**
Chronic kidney diseases	60 (14.02%)	32 (14.04%)	28 (14.00%)	0.992
Chronic hepatic diseases	48 (11.21%)	31 (13.60%)	17 (8.50%)	0.095
**Bacteremia**	31 (7.24%)	12 (5.26%)	19 (9.50%)	0.092
**Shock**	9 (2.10%)	5 (2.19%)	4 (2.00%)	0.890
Infection type				
Community acquired	62 (14.49%)	18 (7.89%)	44 (22.00%)	**<0.001**
Health care acquired	190 (44.39%)	124 (54.39%)	66 (33.00%)	**<0.001**
Hospital associated	176 (41.12%)	86 (37.72%)	90 (45.00%)	0.127
Type of specimen culture-positive bacteria				
One	346 (80.84%)	188 (82.46%)	158 (79.00%)	0.365
Two	79 (18.46%)	39 (17.11%)	40 (20.00%)	0.441
Three	3 (0.70%)	1 (0.44%)	2 (1.00%)	0.487
Types of bacteria co-infected with KP				
*Escherichia coli (E. coli)*	19 (4.44%)	7 (17.50%)	12 (28.57%)	0.287^**^
*Pseudomonas aeruginosa*	17 (3.97%)	11 (27.50%)	6 (14.29%)	0.140^**^
*Acinetobacter baumannii*	9 (2.10%)	5 (12.50%)	4 (9.52%)	0.666^**^
*Staphylococcus aureus*	18 (4.21%)	6 (15.00%)	12 (28.57%)	0.138^**^
Other bacteria	19 (4.44%)	11 (27.50%)	8 (19.05%)	0.365^**^
**MDR KP**	89 (20.79%)	52 (22.81%)	37 (18.50%)	0.273
CRKP	31 (7.24%)	17 (7.46%)	14 (7.00%)	0.856
ESBL-KP	58 (13.55%)	35 (15.35%)	23 (11.50%)	0.246
**No-MDR KP**	339 (79.21%)	176 (77.19%)	163 (81.50%)	0.842
Use of medical devices				
Urinary catheter	153 (35.75%)	101 (44.30%)	52 (26.00%)	**<0.001**
Nasogastric catheter	108 (25.23%)	67 (29.39%)	41 (20.50%)	**0.035**
T-tube catheter	6 (1.40%)	3 (1.32%)	3 (1.50%)	0.872
Arterial catheter and venous catheter	60 (14.02%)	42 (18.42%)	18 (9.00%)	**0.005**
Mechanical ventilation	98 (22.90%)	63 (27.63%)	35 (17.50%)	**0.013**
Tracheal cannula	100 (23.36%)	59 (25.88%)	41 (20.50%)	0.190
Tracheostomy	32 (7.48%)	15 (6.58%)	17 (8.50%)	0.451
Endoscopy	34 (7.94%)	24 (10.53%)	10 (5.00%)	**0.035**
Bronchoscope	46 (10.75%)	23 (10.09%)	23 (11.50%)	0.638
Hemodialysis	12 (2.80%)	8 (3.51%)	4 (2.00%)	0.345
Peritoneal dialysis	2 (0.47%)	1 (0.44%)	1 (0.50%)	0.926
Other medical devices	98 (22.90%)	65 (28.51%)	33 (16.50%)	**0.003**
Concomitant drug				
Hormone	214 (50.00%)	154 (67.54%)	60 (30.00%)	**<0.001**
Treatment with antibiotics				
**Empirical antibiotic therapy**	257 (60.05%)	157 (68.86%)	100 (50.00%)	**<0.001**
Time to EAT (d)	1 (0, 4)	2.5 (0, 6.75)	0.5 (0, 3)	**<0.001**
Penicillin/third-generation cephalosporins	141 (32.94%)	92 (40.35%)	49 (24.50%)	**<0.001**
Penicillin/third generation cephalosporins + beta-lactamase inhibitor	139 (32.48%)	94 (41.23%)	45 (22.50%)	**<0.001**
Carbapenems	20 (4.67%)	13 (5.70%)	7 (3.50%)	0.282
Quinolones	44 (10.28%)	26 (11.4%)	18 (9.00%)	0.414
Fosfomycin	14 (3.27%)	7 (3.07%)	7 (3.50%)	0.803
Antifungal agent	13 (3.04%)	11 (4.82%)	2 (1.00%)	**0.021**
Single DAT				
beta-lactams	137 (32.01%)	77 (33.77%)	60 (30.00%)	0.404
Quinolones	27 (6.31%)	14 (6.14%)	13 (6.50%)	0.879
Others	9 (2.10%)	4 (1.75%)	5 (2.50%)	0.592
Combined DAT				
Combination of two antimicrobials	61 (14.25%)	31 (13.60%)	30 (15.00%)	0.679
Penicillin/third generation cephalosporins + quinolones/aminodycosides	9 (2.10%)	2 (0.88%)	7 (3.50%)	0.059
Penicillin/third-generation cephalosporins + beta-lactamase inhibitor + quinolones/aminodycosides	21 (4.91%)	12 (5.26%)	9 (4.50%)	0.715
Combination of ≥ triple antimicrobials	27 (6.31%)	23 (10.09%)	4 (2.00%)	**0.001**
**Time to irrational use of antibiotics**	0 (0, 2)	0 (0, 3)	0 (0, 1)	**0.010**
**Time to rational use of antibiotics**	2 (0, 7)	3 (1, 8.75)	1 (0, 5)	**<0.001**
Microbiological clearance failure	155 (36.21%)	66 (28.95%)	89 (44.50%)	**0.001**
Clinical outcomes				
Clinical stability	141 (32.94%)	82 (35.96%)	59 (29.50%)	0.156
On the mend	82 (19.16%)	28 (12.28%)	54 (27.00%)	**<0.001**
Deterioration	4 (0.93%)	3 (1.32%)	1 (0.50%)	0.382
Automatic discharge or transfer	81 (18.93%)	38 (16.67%)	43 (21.50%)	0.203
Readmission	72 (16.82%)	50 (21.93%)	22 (11.00%)	**0.003**
Reinfection	9 (2.10%)	5 (2.19%)	4 (2.00%)	0.890
LOS	11 (7, 19.25)	13 (8, 22)	9 (6, 15)	**<0.001**
LOS after infection	5 (2, 11)	7 (3, 12)	4 (2, 10)	**0.007**
Direct admission to ICU	22 (5.14%)	9 (3.95%)	13 (6.50%)	0.233
LOS in ICU	0 (0, 0)	0 (0, 0)	0 (0, 0)	0.795
In-hospital mortality	38 (8.88%)	22 (9.65%)	16 (8.00%)	0.550
30-day mortality	72 (16.82%)	42 (18.42%)	30 (15.00%)	0.345
90-day mortality	86 (20.09%)	53 (23.25%)	33 (16.50%)	0.082
Total mortality	101 (23.60%)	64 (28.07%)	37 (18.50%)	**0.020**

*:*P* < 0.05. **: Co-infected bacteria in immunocompromised patients (n=40), Co-infected bacteria in immunocompetent patients (n=42). Fever≥72H: temperature at or above 38 degrees Celsius. qSOFA, quick sepsis-related organ failure assessment; ADL, activity of daily living score; aCCI, age-adjusted charlson comorbidity index; ESBL, extended-spectrum beta-lactamases; CRKP, carbapenem-resistant KP; MDR, multidrug-resistant; EAT, empirical antibiotic therapy; DAT, definite antibiotic therapy; LOS, length of stay; ICU, intensive care unit.Bold values indicate significant differences (P<0.05).

### 3.2 Microbiological and biochemical characteristics

**Figure 4:**
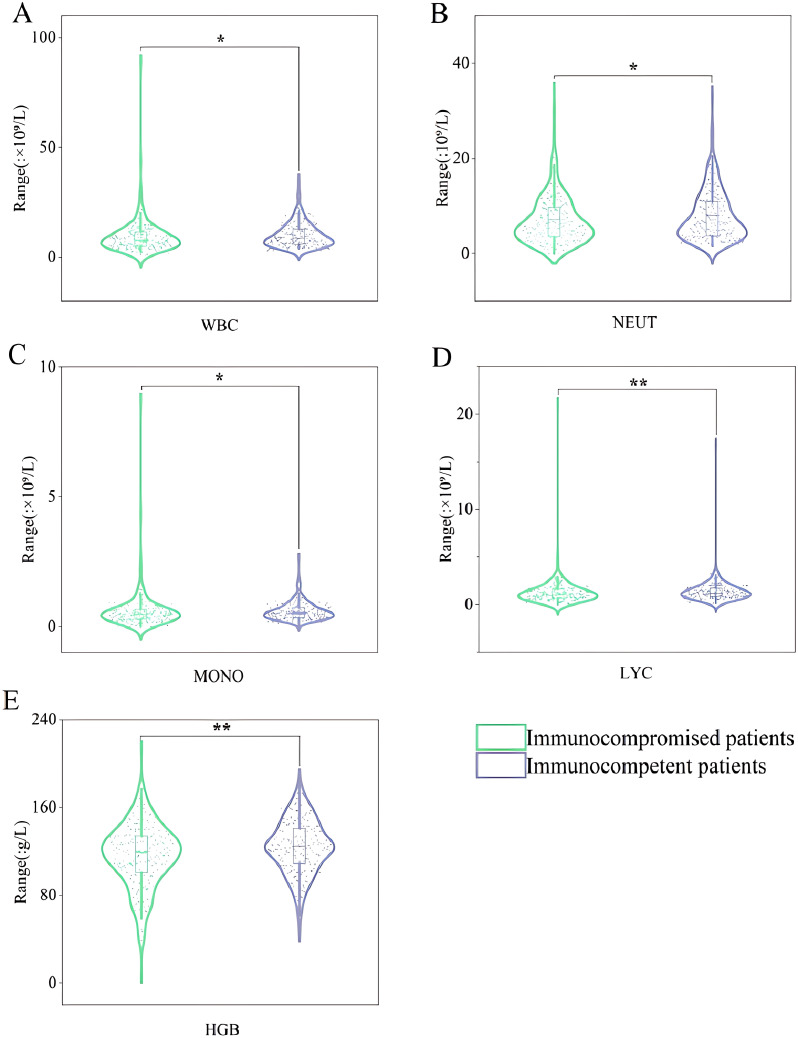
Violin box plots of laboratory test indicators. **(A)**: WBC. **(B)**: NEUT. **(C)**: MONO. **(D)**: LYC. **(E)**: HGB. "*" represented that *P*-value for comparison of immunocompromised patients and immunocompetent patients are less than 0.05. "**" represented that P-value for comparison of immunocompromised patients and immunocompetent patients are less than 0.01. WBC, white blood cell; NEUT, neutrophil granulocyte count; MONO, mononuclear granulocyte count; LYC, lymphocyte count; HGB, haemoglobin.

Microbiological tests were performed in all the patients included (**Table 2**). Approximately 80.84% of samples (346/428) were monomicrobial, and 19.16% (82/428) were polymicrobial. Among other organisms detected along with KP in the samples, *Pseudomonas aeruginosa* and *Acinetobacter baumannii*, as well as fungi and viruses, were more frequently detected in immunocompromised patients. The ESBL-KP isolates accounted for 13.55% (58/428) of all the strains, and all the ESBL-KP isolates were MDR. Immunocompromised patients had a higher infection rate of MDR-KP than immunocompetent patients (22.81% vs. 18.5%). Additionally, WBC (*P* = 0.013), NEUT (*P* = 0.045), MONO (*P* = 0.025), LYC (*P* = 0.008), and HGB (*P* = 0.004) were lower in immunocompromised patients than those in immunocompetent patients (**Figure 4**). However, no difference was observed in PLT, NLR, PLR, ALT, AST and CR between the two groups, as shown in **Supplementary Figure S1**.

### 3.3 Treatment and outcomes

By comparing the invasive procedures, hormonal drug use, antimicrobial strategies, and clinical outcomes between the two groups (**Table 2**), in the use of urinary catheters (*P* = 0.001), nasogastric catheters (*P* = 0.035), arterial catheters and venous catheters (*P* = 0.005), mechanical ventilation (*P* = 0.013), endoscopy (*P* = 0.003), and other medical devices (*P* = 0.003) was found higher in the immunocompromised group. The rates of hormonal drug use (*P* < 0.001), empirical antibiotic therapy (EAT) (*P* < 0.001), time to EAT (*P* < 0.001), use of penicillin/third-generation cephalosporins in EAT (*P* < 0.001), use of penicillin/third-generation cephalosporins combined with beta-lactamase inhibitors in EAT (*P* < 0.001), use of antifungal agent in EAT (*P* < 0.021), and combination of more than three antimicrobials in EAT (*P* = 0.001) were also significantly higher in the immunocompromised group. The length of irrational (*P* = 0.01) and rational (*P* < 0.001) use of antibiotics was longer in immunocompromised patients than those in immunocompetent patients, but the rate of microbiological clearance failure was higher in the immunocompetent group (*P* = 0.001). The rate of improvement was higher in the immunocompetent group (*P* < 0.001), while the rate of readmission was higher in immunocompromised patients (*P* = 0.003). Besides, LOS (*P* < 0.001), LOS after infection (*P* = 0.007), and total mortality (*P* = 0.02) were all higher in the immunocompromised group.

### 3.4 Risk factors associated with total mortality in immunocompromised patients

We performed a multivariable analysis to identify the risk factors for mortality. In univariate analysis, fever at discharge, aCCI, mechanical ventilation, etc., were associated with mortality. These variables were then included in a multiple logistic regression model. Independent risk factors for mortality in immunocompromised patients were aCCI (OR: 1.292, 95%CI: 1.086-1.537, *P* < 0.004) and microbiological clearance failure (OR: 4.175, 95%CI: 1.966-8.866, *P* < 0.001) (**Table 3**).

**Table 3:** Risk factors for mortality in immunocompromised patients with KP.

Variables	Univariate		Multivariate	
	*P*-value*	OR (95%CI)	*P*-value*	OR (95%CI)
**Sex, M**	0.220	0.659 (0.339-1.283)		
**Age, y>65**	**0.009**	2.566 (1.271-5.184)	0.516	1.358 (0.539-3.421)
Fever at time of admission	0.382	1.367 (0.678-2.756)		
Fever at time of discharge	**0.003**	0.215 (0.079-0.582)	0.473	1.578 (0.454-5.485)
History				
Drinking	0.723	1.202 (0.436-3.311)		
Smoking	0.615	0.793 (0.321-1.958)		
Surgical operation	0.311	1.350 (0.755-2.414)		
Hospitalization	0.487	0.812 (0.451-1.462)		
Infection	0.598	1.187 (0.628-2.242)		
Scores				
ADL	**0.004**	0.987 (0.979-0.996)	0.704	0.997 (0.985-1.011)
aCCI	**<0.001**	1.267 (1.115-1.439)	**0.004**	1.292 (1.086-1.537)
qSOFA ≥ 2	0.102	3.578 (0.778-16.457)		
Comorbid conditions				
Nervous system diseases	**0.026**	1.990 (1.087-3.643)	0.575	1.255 (0.567-2.779)
Respiratory diseases	0.192	1.474 (0.823-2.641)		
Cardiovascular diseases	0.065	1.733 (0.967-3.108)		
Digestive diseases	0.896	0.952 (0.456-1.989)		
History of solid organ tumor	0.952	0.959 (0.246-3.735)		
Chronic kidney diseases	0.677	0.833 (0.353-1.965)		
Chronic hepatic diseases	0.898	1.056 (0.458-2.436)		
Infection type				
Community acquired	0.271	0.489 (0.137-1.748)		
Health care acquired	0.954	1.017 (0.569-1.917)		
Hospital associated	0.572	1.186 (0.656-2.143)		
MDR KP type				
ESBL-KP	0.197	1.645 (0.772-3.506)		
CRKP	0.898	1.073 (0.362-3.179)		
Use of medical devices				
Urinary catheter	0.625	1.156 (0.647-2.064)		
Nasogastric catheter	0.095	1.688 (0.914-3.120)		
Arterial catheter and venous catheter	0.646	1.187 (0.572-2.460)		
Mechanical ventilation	**0.003**	2.595 (1.398-4.817)	0.138	1.788 (0.829-3.857)
Tracheal cannula	0.069	1.798 (0.955-3.385)		
Tracheostomy	0.640	1.305 (0.428-3.979)		
Endoscopy	0.197	0.480 (0.157-1.464)		
Bronchoscope	**0.001**	4.822 (1.968-11.814)	0.474	0.623 (0.170-2.277)
Hemodialysis	0.549	1.564 (0.363-6.744)		
Other medical devices	0.936	0.974 (0.513-1.850)		
**Hormone**	0.101	0.605 (0.331-1.104)		
**Empirical antibiotic therapy**	0.982	0.993 (0.532-1.852)		
Time to EAT (d)	0.281	1.020 (0.984-1.057)		
Penicillin/third-generation cephalosporins	0.397	0.772 (0.425-1.404)		
Penicillin/third generation cephalosporins + beta-lactamase inhibitor	**0.049**	1.797 (1.003-3.222)	0.404	1.439 (0.612-3.380)
Carbapenems	0.395	1.653 (0.520-5.255)		
Quinolones	0.250	0.409 (0.089-1.879)		
Fosfomycin	0.976	1.026 (0.194-5.427)		
Antifungal agent	0.199	2.232 (0.656-7.589)		
Single DAT				
beta-lactams	0.261	0.696 (0.370-1.309)		
Quinolones	0.250	0.409 (0.089-1.879)		
Others	0.553	1.731 (0.282-10.610)		
Combined DAT				
Combination of two antimicrobials	0.094	1.645 (0.919-2.947)		
Combination of ≥ triple antimicrobials	0.009	3.210 (1.336-7.712)	0.183	2.055 (0.712-5.930)
**Time to irrational use of antibiotics**	0.017	1.056 (1.010-1.104)	0.241	1.032 (0.979-1.088)
**Time to rational use of antibiotics**	0.657	0.992 (0.956-1.028)		
**Microbiological clearance failure**	**<0.001**	**4.675 (2.503-8.731)**	**<0.001**	**4.175 (1.966-8.866)**
LOS	0.212	1.008 (0.996-1.021)		
LOS after infection	0.719	1.003 (0.985-1.022)		
Direct admission to ICU	0.076	3.390 (0.880-13.053)		
LOS in ICU	0.642	0.983 (0.916-1.056)		

*: *P* < 0.05. qSOFA, quick sepsis-related organ failure assessment; ADL, activity of daily living score; aCCI, age-adjusted charlson comorbidity index; ESBL, extended-spectrum beta-lactamases; CRKP, carbapenem-resistant KP; MDR, multidrug-resistant; EAT, empirical antibiotic therapy; DAT, definite antibiotic therapy; LOS, length of stay; ICU, intensive care unit.Bold values indicate significant differences (P<0.05).

### 3.5 Comparison of clinical characteristics of ESBL-KP, CRKP, and non-MDR groups in the study population

As shown in **Table 4**, infection types differed significantly among the three patient subgroups (HCAI: 46.55% vs. 77.42% vs. 41%, *P* < 0.001) (HAI: 12.9% vs. 45.13%, *P* = 0.001). CRKP-infected patients had more severe clinical conditions, with lower median ADL scores (10 vs. 65; *P* < 0.001) and higher qSOFA scores (*P* = 0.001) compared to non-MDR-KP infected patients. They also had higher prevalence of hospitalization history (77.42% vs. 53.69%, *P* = 0.011) and comorbidities such as nervous system diseases (54.84% vs. 30.97%, *P* = 0.007) and respiratory diseases (70.97% vs. 43.36%, *P* = 0.003). Notably, CRKP-infected patients required significantly more invasive procedures: urinary catheters (58.06% vs. 32.45%, *P* < 0.004), nasogastric catheters (51.61% vs. 21.83%, *P* = 0.001), arterial catheter and venous catheter (48.39% vs. 10.03%, *P* = 0.001), mechanical ventilation (58.06% vs. 19.17%, *P* = 0.001), tracheal cannula (54.84% vs. 20.65%, *P* = 0.001), tracheostomy (32.26% vs. 5.01%, *P* = 0.001), and bronchoscopes (38.71% vs. 6.78%, *P* < 0.001). They were more likely to receive combination antibiotic regimens (19.35% vs. 4.13%, *P* = 0.002) and had a higher rate of microbiological clearance failure (64.52% vs. 33.33%, *P* = 0.001), along with longer hospital stays in ICU (*P* < 0.001).

**Table 4:** Demographic characteristics, clinical characteristics, and outcomes in all patients (ESBL-KP vs. CRKP vs. non-MDR).

Variables	ESBL-KP (n=58)	CRKP (n=31)	non-MDR (n=339)	*P*-value*
**Sex, M**	45 (77.59%)	24 (77.42%)	223 (65.78%)	0.269
Age group, y				
0-65	17 (19.31%)	9 (29.03%)	137 (40.41%)	0.153
>65	41 (70.69%)	22 (70.97%)	202 (59.59%)	0.153
Fever ≥ 72H				
Time of admission	12 (20.69%)	10 (32.26%)	50 (14.75%)	0.031
Time of discharge	5 (8.62%)	5 (16.13%)	13 (3.83%)	0.024
No more fever after treatment	8 (13.79%)	6 (19.35%)	42 (12.39%)	0.570
Infection type				
Community acquired	12 (20.69%)	3 (9.68%)	47 (13.86%)	0.305
Health care acquired	27 (46.55%)	24 (77.42%)	139 (41.00%)	**<0.001*^bc^***
Hospital associated	19 (32.76%)	4 (12.90%)	153 (45.13%)	**0.001*^c^***
History				
Drinking	7 (12.07%)	3 (9.68%)	33 (9.73%)	0.865
Smoking	4 (6.90%)	4 (12.90%)	38 (11.21%)	0.541
Surgical operation	39 (67.24%)	17 (54.84%)	132 (38.94%)	**<0.001*^a^***
Hospitalization	44 (75.86%)	24 (77.42%)	182 (53.69%)	**0.001*^cd^***
Infection	42 (72.41%)	25 (80.65%)	219 (64.60%)	0.120
Scores				
ADL	45 (20, 71.5)	10 (0, 40)	65 (20, 95)	**<0.001*^c^***
aCCI	4 (3, 6)	4 (2, 5)	3 (2, 5)	0.038
qSOFA	0 (0, 1)	1 (0, 1)	0 (0, 1)	**0.001*^c^***
Comorbid conditions				
Nervous system diseases	24 (41.38%)	17 (54.84%)	105 (30.97%)	**0.012*^c^***
Respiratory diseases	30 (51.72%)	22 (70.97%)	147 (43.36%)	**0.009*^c^***
Cardiovascular diseases	30 (51.72%)	16 (70.97%)	142 (41.89%)	0.253
Digestive diseases	11 (18.97%)	8 (51.61%)	54 (15.93%)	0.344
History of solid organ tumor	5 (8.62%)	1 (25.81%)	6 (1.77%)	0.044
Chronic kidney disease	11 (18.97%)	5 (16.13%)	44 (12.98%)	0.472
Chronic hepatic diseases	8 (13.79%)	4 (12.90%)	36 (10.62%)	0.751
Use of medical device				
Urinary catheter	25 (43.10%)	18 (58.06%)	110 (32.45%)	**0.008*^c^***
Nasogastric catheter	18 (31.03%)	16 (51.61%)	74 (21.83%)	**0.001*^c^***
T-tube catheter	1 (1.72%)	1 (3.23%)	4 (1.18%)	0.584
Arterial catheter and venous catheter	11 (18.97%)	15 (48.39%)	34 (10.03%)	**<0.001*^bc^***
Mechanical ventilation	15 (25.86%)	18 (58.06%)	65 (19.17%)	**<0.001*^bc^***
Tracheal cannula	13 (22.41%)	17 (54.84%)	70 (20.65%)	**<0.001*^bc^***
Tracheostomy	5 (8.62%)	10 (32.26%)	17 (5.01%)	**<0.001*^bc^***
Endoscopy	8 (13.79%)	2 (6.45%)	24 (7.08%)	0.256
Bronchoscope	11 (18.97%)	12 (38.71%)	23 (6.78%)	**<0.001*^cd^***
Hemodialysis	2 (3.45%)	0 (0.00%)	10 (2.95%)	0.960
Peritoneal dialysis	0 (0.00%)	0 (0.00%)	2 (0.59%)	0.490
Other medical devices	10 (17.24%)	7 (22.58%)	81 (23.89%)	0.537
Treatment with antibiotics				
**Empirical Antibiotic Therapy**	42 (72.41%)	15 (48.39%)	200 (59.00%)	0.537
Time to EAT (d)	3 (0, 7.25)	0 (0, 4)	1 (0, 3)	**0.002*^d^***
Penicillin/third-generation cephalosporins	24 (41.38%)	7 (22.58%)	110 (32.45%)	0.182
Penicillin/third-generation cephalosporins + beta-lactamase inhibitor	25 (43.10%)	12 (38.71%)	102 (30.09%)	0.110
Carbapenems	4 (6.90%)	5 (16.13%)	11 (3.24%)	0.018
Quinolones	9 (15.52%)	3 (9.68%)	32 (9.44%)	0.407
Fosfomycin	8 (13.79%)	0 (0.00%)	6 (1.77%)	**<0.001*^d^***
Antifungal agent	5 (8.62%)	3 (9.68%)	5 (1.47%)	**0.001*^d^***
Single DAT				
beta-lactams	16 (27.59%)	6 (19.35%)	115 (33.92%)	0.185
Quinolones	2 (3.45%)	0 (0.00%)	25 (7.37%)	0.060
Others	4 (6.90%)	1 (3.23%)	4 (1.18%)	**0.005*^a^***
Combined DAT				
Combination of two antimicrobials	14 (24.14%)	4 (12.90%)	43 (12.68%)	0.151
Combination of ≥ triple antimicrobials	7 (12.07%)	6 (19.35%)	14 (4.13%)	**0.003*^c^***
**Time to irrational use of antibiotics**	2 (0, 6)	0 (0, 2)	0 (0, 1)	**<0.001*^d^***
**Time to rational use of antibiotics**	2.5 (1, 8.25)	1 (0, 11)	2 (0, 7)	0.522
**Microbiological clearance failure**	22 (37.93%)	20 (64.52%)	113 (33.33%)	**0.002*^bc^***
Concomitant drugs				
Hormone	28 (48.28%)	17 (54.84%)	169 (49.85%)	0.834
Clinical outcomes				
Clinical stability	18 (31.03%)	10 (32.26%)	113 (33.33%)	0.939
On the mend	7 (12.07%)	4 (12.90%)	71 (20.94%)	0.186
Deterioration	0 (0.00%)	1 (3.23%)	3 (0.88%)	0.805
Automatic discharge or transfer	9 (15.52%)	10 (32.26%)	62 (18.29%)	0.127
Readmission	14 (24.14%)	0 (0.00%)	58 (17.11%)	**0.014*^b^***
Reinfection	3 (5.17%)	0 (0.00%)	6 (1.77%)	0.165
LOS	12.5 (7.75, 24.25)	1 (0, 11)	2 (0, 7)	**0.008*^d^***
LOS after infection	6.5 (2, 14.25)	5 (1, 17)	5 (2, 10)	0.866
Direct admission to ICU	5 (8.62%)	7 (22.58%)	9 (2.65%)	**<0.001*^c^***
LOS in ICU	0 (0, 0)	3 (0, 17)	0 (0, 0)	**<0.001*^bcd^***
In-hospital mortality	5 (8.62%)	7 (22.58%)	26 (7.67%)	0.043
30-day mortality	10 (17.24%)	8 (25.81%)	54 (15.93%)	0.370
90-day mortality	13 (22.41%)	9 (29.03%)	64 (18.88%)	0.359
Total mortality	18 (31.03%)	11 (35.48%)	72 (21.24%)	0.072

a: No significant difference (*P* > 0.017) between any of the pairs in *post hoc* analysis.

b: Significantly different (*P* < 0.017) between ESBL-KP and CRKP.

c: Significantly different (*P* < 0.017) between CRKP and Non-MDR.

d: Significantly different (*P* < 0.017) between ESBL-KP and non-MDR. Fever ≥ 72H: temperature at or above 38 degrees Celsius. qSOFA, quick sepsis-related organ failure assessment; ADL, activity of daily living score; aCCI, age-adjusted charlson comorbidity index; EAT, empirical antibiotic therapy; DAT, definite antibiotic therapy; LOS, length of stay; ICU, intensive care unit; ESBL, extended-spectrum beta-lactamases; CRKP, carbapenem-resistant KP; MDR, multidrug-resistant.Bold values indicate significant differences in overall comparisons among three groups (P<0.05).

Compared to non-MDR-KP infected patients, ESBL-KP-infected patients more frequently had surgical history (67.24% vs. 38.94%, *P* < 0.001) and hospitalization history (75.86% vs. 53.69%, *P* = 0.002). Their management more frequently involved bronchoscopic procedures (18.97% vs. 6.78%, *P* = 0.005) and specific antimicrobial regimens, including fosfomycin (13.79% vs. 1.77%, *P* < 0.001) and antifungal agents (8.62% vs. 1.47%, *P* = 0.006) during EAT. Furthermore, these patients experienced extended durations of both empirical (*P* = 0.003) and inappropriate antibiotic administration (*P* < 0.001).

While CRKP and ESBL-KP infections shared many clinical features, some differences were observed. CRKP infections were associated with increased use of invasive procedures: arterial catheter and venous catheter (48.39% vs. 18.97%, *P* = 0.004), mechanical ventilation (58.06% vs. 25.86%, *P* = 0.003), tracheal cannula (54.84% vs. 22.41%, *P* = 0.002), and tracheostomy (32.26% vs. 8.62%, *P* = 0.005). Our study found nearly twice as CRKP-infected patients failed to eradicate microbiologicals compared to ESBL-KP-infected patients (64.52% vs. 37.93%, *P* < 0.017).

### 3.6 Comparison of clinical characteristics of ESBL-KP, CRKP, and non-MDR groups in the study population with different immune states

In the immunocompromised patients of three groups, key indicators such as infection types, surgical or hospitalization history, ADL and qSOFA scores, bronchoscopy usage, triple antimicrobial therapy, microbiological clearance failure, and LOS in ICU exhibited similar distributions to those observed in the overall population. Compared to non-MDR-KP infected patients, more CRKP-infected patients presented with febrile symptoms on admission (47.06% vs.15.91%, *P* = 0.005) and discharge (29.41% vs. 5.11%, *P* = 0.004). ESBL-KP-infected patients had a longer duration of EAT (*P* = 0.003), and were more likely to receive penicillin/third generation cephalosporins combined with beta-lactamase inhibitors (62.86% vs. 35.80%, *P* = 0.003), quinolones (25.71% vs. 7.95%, *P* = 0.005) and fosfomycin (14.29% vs. 1.14%, *P* = 0.002) in EAT compared to non-MDR-KP infected patients (**Table 5**).

**Table 5:** Demographic characteristics, clinical characteristics, and outcomes in immunocompromised patients and immunocompetent patients (ESBL-KP vs. CRKP vs. non-MDR).

Variables	Immunocompromised patients (n=228)				Immunocompetent patients (n=200)			
	ESBL-KP (n=35)	CRKP (n=17)	non-MDR (n=176)	*P*-value*	ESBL-KP (n=23)	CRKP (n=14)	non-MDR (n=163)	*P*-value*
**Sex, M**	26 (74.29%)	14 (82.35%)	121 (68.75%)	0.413	19 (82.61%)	10 (71.43%)	112 (68.71%)	0.359
Age group, y								
0-65	9 (25.71%)	5 (29.41%)	64 (36.36%)	0.436	8 (34.78%)	4 (28.57%)	73 (44.79%)	0.364
>65	26 (74.29%)	12 (70.59%)	112 (63.64%)	0.436	15 (65.22%)	10 (71.43%)	90 (55.21%)	0.364
Fever ≥ 72H								
Time of admission	9 (25.71%)	8 (47.06%)	28 (15.91%)	**0.012*^c^***	3 (13.04%)	2 (14.29%)	22 (13.50%)	0.994
Time of discharge	4 (11.43%)	5 (29.41%)	9 (5.11%)	**0.009*^c^***	1 (4.35%)	0 (0.00%)	4 (2.45%)	0.733
No more fever after treatment	6 (17.14%)	4 (23.53%)	22 (12.50%)	0.425	2 (8.70%)	2 (14.29%)	20 (12.27%)	0.845
Infection type								
Community acquired	3 (8.57%)	1 (5.88%)	14 (7.95%)	0.939	9 (39.13%)	2 (14.29%)	33 (20.25%)	0.119
Health care acquired	17 (48.57%)	15 (88.24%)	92 (52.27%)	**0.013*^bc^***	10 (43.48%)	9 (64.29%)	47 (28.83%)	0.017
Hospital associated	15 (42.86%)	1 (5.88%)	70 (39.77%)	**0.018*^bc^***	4 (17.39%)	3 (21.43%)	83 (50.92%)	**0.002*^d^***
History								
Drinking	2 (5.71%)	1 (5.88%)	16 (9.09%)	0.731	5 (21.74%)	2 (14.29%)	17 (10.43%)	0.336
Smoking	3 (8.57%)	3 (17.65%)	23 (13.07%)	0.619	1 (4.35%)	1 (7.14%)	15 (9.20%)	0.688
Surgical operation	26 (74.29%)	10 (58.82%)	80 (45.45%)	**0.006*^d^***	13 (56.52%)	7 (50.00%)	52 (31.90%)	0.037
Hospitalization	30 (85.71%)	14 (82.53%)	96 (54.55%)	**<0.001*^d^***	14 (60.87%)	10 (71.43%)	86 (52.76%)	0.337
Infection	26 (74.29%)	13 (76.47%)	119 (67.61%)	0.590	16 (69.57%)	12 (85.71%)	100 (61.35%)	0.160
Scores								
ADL	45 (32.5, 70)	10 (10, 40)	70 (27.5, 92.5)	**0.001*^c^***	30 (5, 90)	10 (0, 40)	65 (20, 95)	**<0.001*^c^***
aCCI	5 (3.5, 6)	4 (2, 5)	4 (2, 5)	0.076	3 (2, 5)	4 (2.75, 5)	3 (2, 4)	0.246
qSOFA	0 (0, 0.5)	1 (0, 1)	0 (0, 1)	**0.017*^c^***	0 (0, 1)	1 (0, 1.25)	0 (0, 1)	0.036
Comorbid conditions								
Nervous system diseases	12 (34.29%)	8 (47.06%)	51 (28.98%)	0.279	12 (52.17%)	9 (64.29%)	54 (33.13%)	0.021
Respiratory diseases	17 (48.57%)	12 (70.59%)	87 (49.43%)	0.239	13 (56.52%)	10 (71.43%)	60 (36.81%)	**0.012*^c^***
Cardiovascular diseases	15 (42.86%)	7 (41.18%)	70 (39.77%)	0.941	15 (65.22%)	9 (64.29%)	72 (44.17%)	0.075
Digestive diseases	10 (28.57%)	5 (29.41%)	29 (16.48%)	0.159	1 (4.35%)	3 (21.43%)	25 (15.34%)	0.212
History of solid organ tumor	4 (11.43%)	1 (5.88%)	6 (3.41%)	**0.044*^a^***	1 (4.35%)	0 (0.00%)	0 (0.00%)	**0.010*^a^***
Chronic kidney disease	7 (20.00%)	3 (17.65%)	22 (12.50%)	0.482	4 (17.39%)	2 (14.29%)	22 (13.50%)	0.886
Chronic hepatic diseases	5 (14.29%)	4 (23.53%)	22 (12.50%)	0.494	3 (13.04%)	0 (0.00%)	14 (8.59%)	0.221
Use of medical device								
Urinary catheter	16 (45.71%)	11 (64.71%)	74 (42.05%)	0.196	9 (39.13%)	7 (50.00%)	36 (22.09%)	0.032
Nasogastric catheter	10 (28.57%)	9 (52.94%)	48 (27.27%)	0.105	8 (34.78%)	7 (50.00%)	26 (15.95%)	**0.002*^c^***
T-tube catheter	0 (0.00%)	0 (0.00%)	3 (1.70%)	0.368	1 (4.35%)	1 (7.14%)	1 (0.61%)	0.651
Arterial catheter and venous catheter	6 (17.14%)	7 (41.18%)	29 (16.48%)	0.074	5 (21.74%)	8 (57.14%)	5 (3.07%)	**<0.001*^cd^***
Mechanical ventilation	10 (28.57%)	9 (52.94%)	44 (25.00%)	0.065	5 (21.74%)	9 (64.29%)	21 (12.88%)	**<0.001*^bc^***
Tracheal cannula	6 (17.14%)	9 (52.94%)	44 (25.00%)	**0.027*^a^***	7 (30.43%)	8 (57.14%)	26 (15.95%)	**0.002*^c^***
Tracheostomy	1 (2.86%)	3 (17.65%)	11 (6.25%)	0.182	4 (17.39%)	7 (50.00%)	6 (3.68%)	**<0.001*^c^***
Endoscopy	6 (17.14%)	1 (5.88%)	17 (9.66%)	0.369	2 (8.70%)	1 (7.14%)	7 (4.29%)	0.329
Bronchoscope	6 (17.14%)	6 (35.29%)	11 (6.25%)	**0.002*^c^***	5 (21.74%)	6 (42.86%)	12 (7.36%)	**0.001*^c^***
Hemodialysis	1 (2.86%)	0 (0.00%)	7 (3.98%)	0.608	1 (4.35%)	0 (0.00%)	3 (1.84%)	0.543
Peritoneal dialysis	0 (0.00%)	0 (0.00%)	1 (0.57%)	0.605	0 (0.00%)	0 (0.00%)	1 (0.61%)	0.651
Other medical devices	8 (22.86%)	4 (23.53%)	53 (30.11%)	0.604	2 (8.70%)	3 (21.43%)	28 (17.18%)	0.479
Treatment with antibiotics								
**Empirical Antibiotic Therapy**	28 (80.00%)	11 (64.71%)	118 (67.05%)	0.296	14 (60.87%)	4 (28.57%)	82 (50.31%)	0.160
Time to EAT (d)	5 (2, 10)	1 (0, 8)	2 (0, 5)	**0.011*^d^***	2 (0, 4)	0 (0, 1.5)	1 (0, 3)	0.152
Penicillin/third generation cephalosporins	17 (48.57%)	5 (29.41%)	70 (39.77%)	0.396	7 (30.43%)	2 (14.29%)	40 (24.54%)	0.521
Penicillin/third generation cephalosporins+ beta-lactamase inhibitor	22 (62.86%)	9 (52.94%)	63 (35.80%)	**0.007*^d^***	3 (13.04%)	3 (21.43%)	39 (23.93%)	0.465
Carbapenems	3 (8.57%)	4 (23.53%)	6 (3.41%)	0.051	1 (4.35%)	1 (7.14%)	5 (3.07%)	0.603
Quinolones	9 (25.71%)	3 (17.65%)	14 (7.95%)	**0.015*^d^***	0 (0.00%)	0 (0.00%)	18 (11.04%)	0.021
Fosfomycin	5 (14.29%)	0 (0.00%)	2 (1.14%)	**<0.001*^d^***	3 (13.04%)	0 (0.00%)	4 (2.45%)	0.024
Antifungal agent	4 (11.43%)	2 (11.76%)	5 (2.84%)	**0.015*^a^***	1 (4.35%)	1 (7.14%)	0 (0.00%)	**0.010*^a^***
Single DAT								
beta-lactams	9 (25.71%)	2 (11.76%)	66 (37.50%)	0.055	7 (30.43%)	4 (28.57%)	49 (30.06%)	0.922
Quinolones	2 (5.71%)	0 (0.00%)	12 (6.82%)	0.316	0 (0.00%)	0 (0.00%)	13 (7.98%)	0.093
Others	2 (5.71%)	0 (0.00%)	2 (1.14%)	0.091	2 (8.70%)	1 (7.14%)	2 (1.23%)	0.172
Combined DAT								
Combination of two antimicrobials	7 (20.00%)	4 (23.53%)	22 (12.50%)	0.310	7 (30.43%)	2 (14.29%)	21 (12.88%)	0.129
Combination of ≥ triple antimicrobials	6 (17.14%)	5 (29.41%)	12 (6.82%)	**0.012*^c^***	1 (4.35%)	1 (7.14%)	2 (1.23%)	0.172
**Time to irrational use of antibiotics**	4 (0, 8)	0 (0, 2)	0 (0, 2)	**<0.001*^d^***	2 (0, 4)	0 (0, 1.5)	1 (0, 3)	0.152
**Time to rational use of antibiotics**	3 (1, 11)	5 (0, 13)	3 (1, 7)	0.671	0 (0, 3)	0 (0, 0.75)	0 (0, 1)	0.162
**Microbiological clearance failure**	13 (37.14%)	10 (58.82%)	43 (24.43%)	**0.009*^c^***	9 (39.13%)	10 (71.43%)	70 (42.94%)	0.103
Concomitant drugs								
Hormone	22 (62.86%)	13 (76.47%)	119 (67.61%)	0.616	6 (26.09%)	4 (28.57%)	50 (30.67%)	0.895
Clinical outcomes								
Clinical stability	12 (34.29%)	6 (35.29%)	64 (36.36%)	0.971	6 (26.09%)	4 (28.57%)	49 (30.06%)	0.922
On the mend	1 (2.86%)	2 (11.76%)	25 (14.20%)	0.100	6 (26.09%)	2 (14.29%)	46 (28.22%)	0.487
Deterioration	0 (0.00%)	1 (5.88%)	2 (1.14%)	0.909	0 (0.00%)	0 (0.00%)	1 (0.61%)	0.661
Automatic discharge or transfer	4 (11.43%)	4 (23.53%)	30 (17.05%)	0.521	5 (21.74%)	6 (42.86%)	32 (19.63%)	0.170
Readmission	11 (31.43%)	0 (0.00%)	39 (22.16%)	**0.006*^b^***	3 (13.04%)	0 (0.00%)	19 (11.66%)	0.180
Reinfection	2 (5.71%)	0 (0.00%)	3 (1.70%)	0.200	1 (4.35%)	0 (0.00%)	3 (1.84%)	0.543
LOS	13 (8.5, 24.5)	17 (9, 39)	12 (8, 21)	0.208	10 (5, 20)	19.5 (8.25, 22.75)	9 (6, 14)	0.059
LOS after infection	8 (2, 13)	5 (1, 17)	7 (3, 12)	0.981	6 (1, 17)	5.5 (0.75, 14)	4 (2, 9)	0.738
Direct admission to ICU	1 (2.86%)	3 (17.65%)	5 (2.84%)	0.471	4 (17.39%)	5 (35.71%)	4 (2.45%)	**0.001*^cd^***
LOS in ICU	0 (0, 0)	0 (0, 13)	0 (0, 0)	**<0.001*^bc^***	0 (0, 3)	8 (0, 22.5)	0 (0, 0)	**<0.001*^bcd^***
In-hospital mortality	4 (11.43%)	4 (23.53%)	14 (7.95%)	0.171	1 (4.35%)	3 (21.43%)	12 (7.36%)	0.220
30-day mortality	8 (22.86%)	4 (23.53%)	30 (17.05%)	0.626	2 (8.70%)	4 (28.57%)	24 (14.72%)	0.285
90-day mortality	10 (28.57%)	4 (23.53%)	39 (22.16%)	0.723	3 (13.04%)	5 (35.71%)	25 (15.34%)	0.184
Total mortality	13 (37.14%)	5 (29.41%)	46 (26.14%)	0.428	5 (21.74%)	6 (42.86%)	26 (15.95%)	0.071

a: No significant difference (*P* > 0.017) between any of the pairs in *post hoc* analysis.

b: Significantly different (*P* < 0.017) between ESBL-KP and CRKP.

c: Significantly different (*P* < 0.017) between CRKP and non-MDR.

d: Significantly different (*P* < 0.017) between ESBL-KP and non-MDR. Fever≥72H: temperature at or above 38 degrees Celsius. qSOFA, quick sepsis-related organ failure assessment; ADL, activity of daily living score; aCCI, age-adjusted charlson comorbidity index; EAT, empirical antibiotic therapy; DAT, definite antibiotic therapy; LOS, length of stay; ICU, intensive care unit; ESBL, extended-spectrum beta-lactamases; CRKP, carbapenem-resistant KP; MDR, multidrug-resistant.Bold values indicate significant differences in overall comparisons among three groups (P<0.05).

In the immunocompetent population, the need for mechanical ventilation was higher in the CRKP group than that in the ESBL-KP group (64.29% vs. 21.74%, *P* = 0.015). ESBL-KP-infected patients received more arterial catheter and venous catheter placements (21.74% vs. 3.07%, *P* = 0.001) and ICU admissions (17.39% vs. 2.45%, *P* = 0.001) than non-MDR-KP-infected patients. The duration of ICU stays in the CRKP and ESBL-KP groups exceeded that in the non-MDR group (*P* < 0.001) (*P* = 0.002) (**Table 5**).

**Figure 5:**
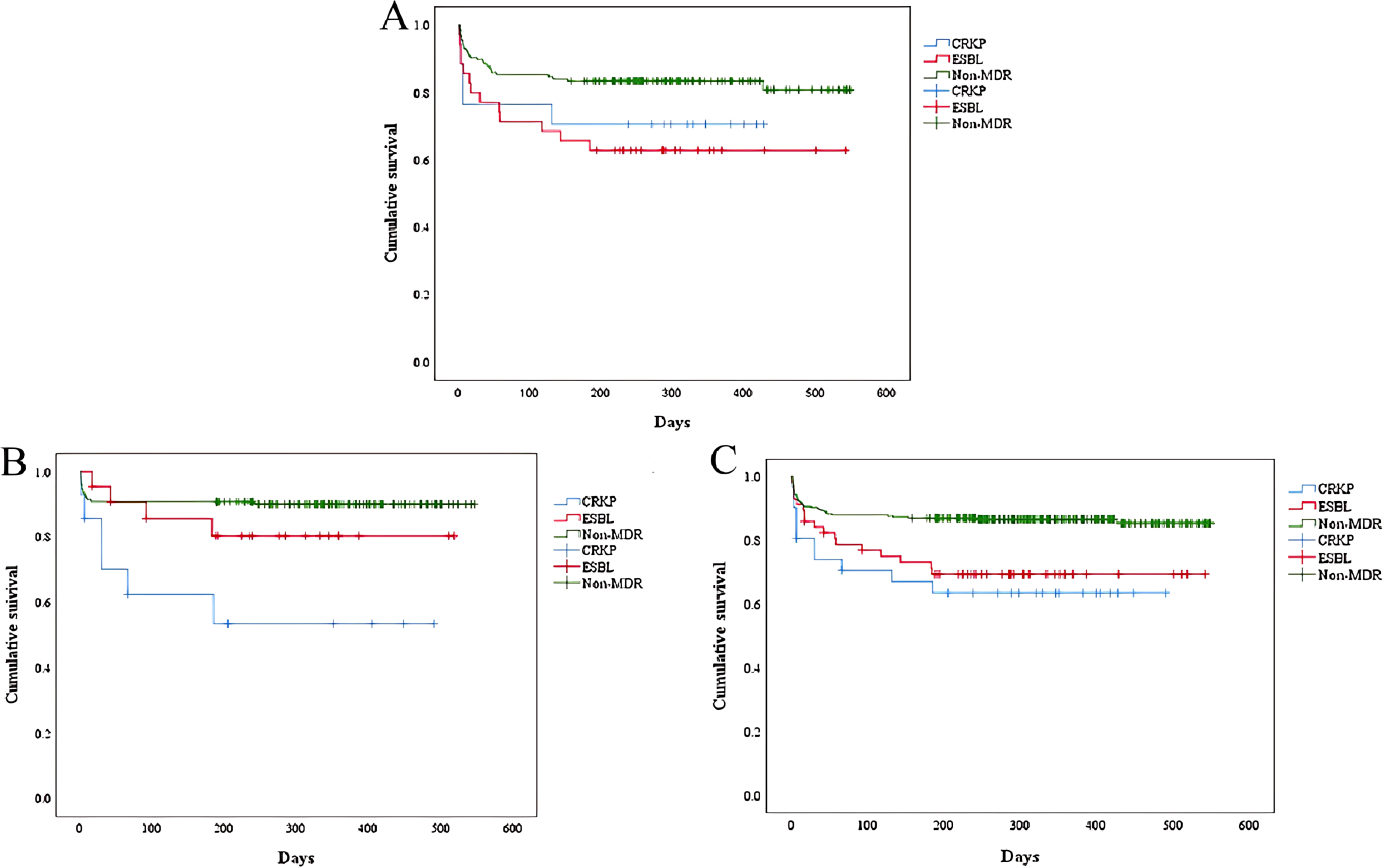
Kaplan-Meier curves compared the cumulative survival of patients (ESBL-KP vs. CRKP vs. non-MDR). **(A)** immunocompromised patients. Log rank *P* = 0.0018. **(B)** immunocompetent patients. Log rank *P* = 0.001. **(C)** all the patients. Log rank *P*< 0.001. ESBL, extended-spectrum beta-lactamases; CRKP, carbapenem-resistant KP; MDR, multidrug-resistant.

Cumulative survival rates for the three groups are demonstrated in **Figure 5**. It was observed that the ESBL-KP group had the lowest cumulative survival in immunocompromised patients. In contrast, among all patients and immunocompetent patients, the CRKP group had the lowest cumulative survival rates, trailed by the ESBL-KP group.

## 4 Discussion

A substantial number of patients hospitalized for KP infection were immunocompromised for a variety of reasons, the most common of which were the use of immunosuppressive drugs and being in the active stage of malignancy. Similar to the results of previous studies, 30.7% of immunocompromised patients had more than one underlying condition (Di Pasquale et al., 2019; Ramirez et al., 2020; Mohd Asri et al., 2021; Liu et al., 2023). An association between underlying conditions has been proposed (Di Pasquale et al., 2019), but which underlying condition has the greatest effect on immunocompromise remains unclear, necessitating mechanistic investigations into their hierarchical impacts.

In our study, compared to immunocompetent patients, immunocompromised patients underwent more invasive procedures, including urinary catheters, nasogastric catheters, arterial catheters, venous catheters, mechanical ventilation and endoscopy, which was consistent with previous research (Di Pasquale et al., 2019; Mohd Asri et al., 2021; Liu et al., 2023). These invasive procedures disrupt the body’s natural defense barriers and thereby increase the risk of infections in immunocompromised patients (Guo et al., 2024). Compared to other invasive procedures, nasogastric catheters directly contact the respiratory tract, and patients may receive relatively few infection prevention measures. This may be the reason that the respiratory tract serves as the primary route for KP infection in immunocompromised patients. As mentioned in many studies (Chen et al., 2022; Kong et al., 2025), invasive procedures was a risk factor for mortality in KP-infected patients. However, in our study, invasive procedures was not a significant risk factor. Through multivariate logistic regression analyses, we identified independent risk factors for death in immunocompromised patients with KP, including aCCI score and microbiological clearance failure, which was partly consistent with other reports (Huang et al., 2023).

Interestingly, the rate of microbiological clearance failure in immunocompromised patients was lower than that in immunocompetent patients. A possible explanation is that immunocompromised patients have a more aggressive clinical intervention strategy – they had a significantly higher use of EAT (mainly piperacillin/third-generation cephalosporins) (Hyun et al., 2024). Carbapenems seemed not to be the first choice unless the results of drug sensitivity tests were available, similar to a study in Guangdong, China (Huang et al., 2023), but contrary to the findings of another study in Beijing (Liu et al., 2021), which suggests the diversity of antimicrobial patterns in different regions. Combination therapy with over two antibiotics was used more frequently in immunocompromised population. To our knowledge, it has seldom been discussed in prior studies. Given the variety of antimicrobial therapies included in this study, our ability to detect differences in outcomes based on treatment was limited. Since most of the immunosuppressants are corticosteroid, immunocompromised patients were more likely to use hormonal drugs than immunocompetent patients. Corticosteroids may have negative impacts on infection control and eventually shorten the life span of patients (Chen et al., 2022), indirectly posing the high mortality rate in immunocompromised patients with KP. A Meta-analysis suggested KP infections were associated with corticosteroid therapies (Lin et al., 2023).

As mentioned above, active malignancies were one of the most important risk factors for immunocompromised populations. High NLR and PLR levels were factors affecting prognosis for patients with malignancy (Zhang et al., 2024). Although not statistically significant, NLR and PLR levels were higher in immunocompromised patients than those in immunocompetent patients. We also found that WBC, NEUT, MONO, LYC, and HGB levels were lower in immunocompromised patients than those in immunocompetent patients in the first laboratory examination after admission. And the low level of NEUT is evidence that neutropenic patients are well represented in the immunocompromised population (Di Pasquale et al., 2019). Finally, it is necessary to monitor Complete Blood Count (CBC) of patients with different immune function states.

The detection rate of MDR-KP in our study was slightly lower than the average of similar studies (Chen et al., 2022; Liu et al., 2023). Consistent with the results of previous research (Ibrahim, 2023), MDR-KP-infected patients exhibited significantly severe clinical conditions and poorer outcomes compared to non-MDR-KP infected patients. In a comparative study, Tofarides et al. found no significant differences between the ESBL-KP and CRKP groups (Tofarides et al., 2023). However, we found that although both ESBL-KP and CRKP were prevalent within the hospital setting, CRKP were more likely to be acquired through healthcare-associated routes than ESBL-KP, due to the heavier use of invasive procedures. We revealed that CRKP-infected patients usually stay longer in the ICU than ESBL-infected patients. It indicates that prevention and control of the occurrence of CRKP should be focused on ICU. ICU has already been recognized as a factory of creating, disseminating, and amplifying antimicrobial resistance (Chen et al., 2022). Most ICU patients have relatively serious complications and may be treated with longer duration of antibiotics use, which contribute to the induction of carbapenem resistance for KP. Only in immunocompromised patients, ESBL-KP-infected patients had more readmissions than CRKP-infected patients. And, the mortality of ESBL-KP in immunocompromised patients was higher than that of CRKP, which was contrary to the results of former studies (Nguyen et al., 2015; Tofarides et al., 2023). Survival analysis revealed that the vast majority of deaths occurred within 200 days of the infection with KP. During this period, the ESBL-KP group had the lowest cumulative survival in immunocompromised patients, while among both total and immunocompetent patients, the CRKP groups recorded the lowest cumulative survival, trailed by the ESBL-KP groups. This suggested that infection with ESBL-KP had the greatest impact on the survival of immunocompromised patients, but this result could not be ruled out due to the low detection rate of CRKP in our study population.

Our research had some limitations. Firstly, as a retrospective analysis of single-center data, it could not possess extensive representativeness and generalizability. Therefore, we appeal for more clinical centers to participate in this research. Secondly, our study lacked more detailed microbiological data, especially on strain genotypes. Finally, to include as many patient cases as possible and to avoid bias caused by a small sample size, we were conservative in the selection of laboratory examination items and only selected those that were commonly tested upon admission.

In conclusion, our study overcame the limitation of previous research that focused on the respiratory infection by including all samples of KP in our hospital, and revealed the differences among patients in the ESBL-KP group, CRKP group, and non-MDR group with different immune states. Patients infected with KP exhibited significant differences in clinical and microbiological characteristics under various states of immune function, which reminded medical personnel to observe the protocols for invasive procedures and hand hygiene. In clinical treatment, medications should be prescribed in accordance with the results of susceptibility testing and the actual circumstances.

## Data Availability

The original contributions presented in the study are included in the article/**Supplementary Material**. Further inquiries can be directed to the corresponding authors.
