# Supplementary figures and images for: Clinical characteristics, risk factors and prognosis of Klebsiella pneumoniae infection in patients with different states of immune function: a retrospective study
Source: Front Cell Infect Microbiol. 2025 May 30;15:1539554. doi: 10.3389/fcimb.2025.1539554 (PMC12162992; doi:10.3389/fcimb.2025.1539554)

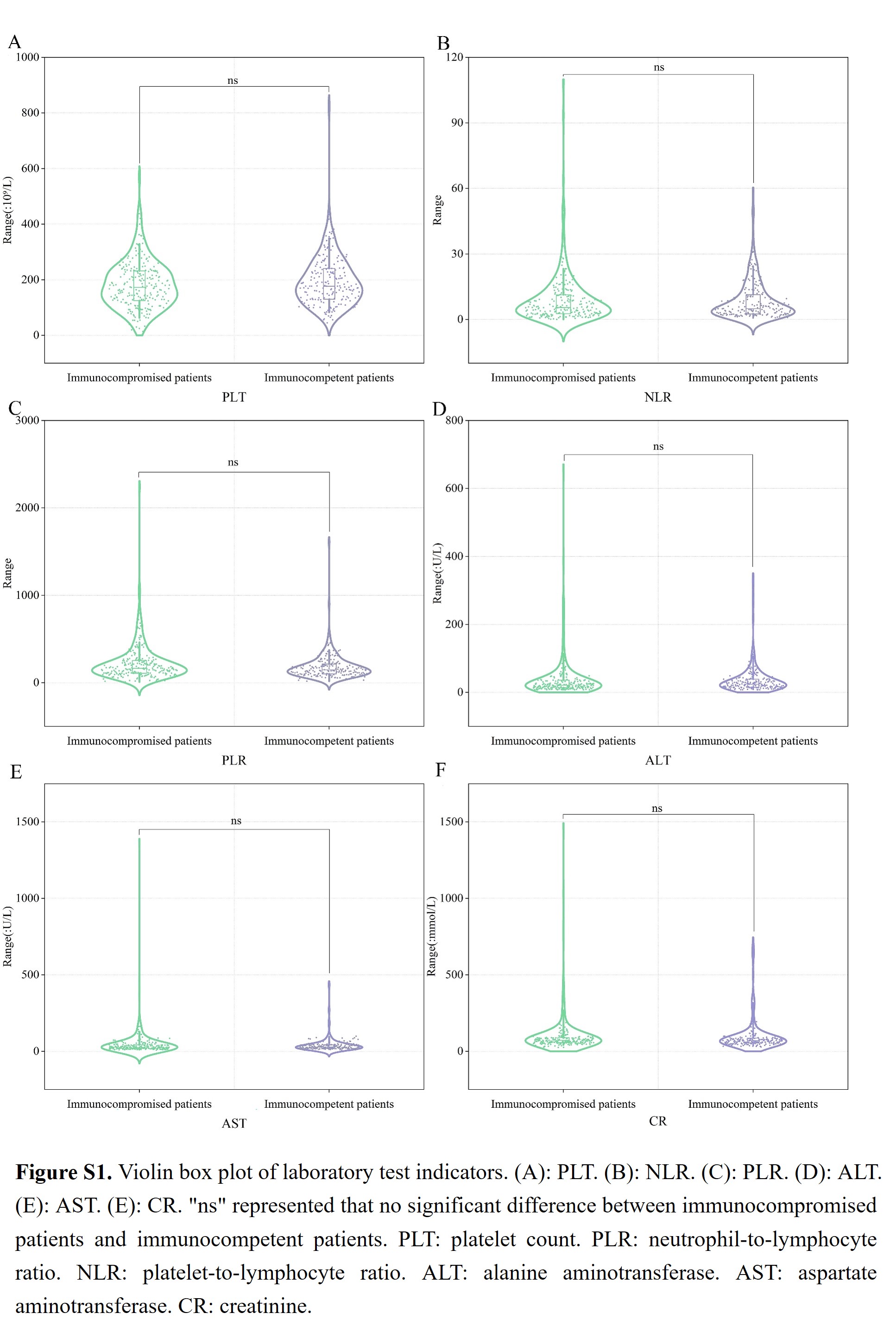

Supplement: Supplementary file 1 [file Image1.jpeg]
